# A Chemically Characterized Extract of *Centipeda minima* Attenuates Inflammation in Macrophages and Alleviates Osteoarthritis Progression in Rats

**DOI:** 10.3390/molecules31203639

**Published:** 2026-10-12

**Authors:** Myong Jin Lee, Young-Joo Kim, Byoung Ha Kim, Shin Jung Park, Hea Sun Chun, Ki Sung Kang

**Affiliations:** 1 College of Korean Medicine, Gachon University, 1342, Seongnamdae-ro, Sujeong-gu, Seongnam 13120, Republic of Korea; myongene@gachon.ac.kr (M.J.L.); smile0341@naver.com (S.J.P.); 2 Institute of Natural Products, Korea Institute of Science and Technology, 679, Saimdang-ro, Gangneung 25451, Republic of Korea; yjkim7801@kist.re.kr; 3 School of Pharmacy, Sungkyunkwan University, Suwon 16419, Republic of Korea; mot37@d-nature.co.kr

**Keywords:** *Centipeda minima*, osteoarthritis, chemically characterized extract

## Abstract

*Centipeda minima*, an edible herb with medicinal properties, contains bioactive sesquiterpene lactones with anti-inflammatory potential, but its nutraceutical application is limited by insufficient standardization. This study evaluated the anti-inflammatory mechanisms of a chemically characterized bioactive-rich extract from *C. minima* (CMXP) and its four isolated active compounds. In LPS-stimulated RAW 264.7 macrophages, CMXP and the four isolated sesquiterpene lactones inhibited nitric oxide (NO) production in a concentration-dependent manner. These inhibitory effects were evident within concentration ranges that did not markedly affect cell viability, whereas reduced cell viability was observed at the highest tested concentrations of CMXP (10 μg/mL), arnicolide C (10 μM), and brevilin A (10 μM). CMXP markedly suppressed iNOS and COX-2 expression, reducing ERK and JNK phosphorylation. In the MIA-induced osteoarthritis rat model, CMXP improved hind limb weight-bearing function, decreased serum IL-6 and cartilage MMP-3 expression, and attenuated subchondral bone loss, as confirmed by micro-computed tomography. CMXP demonstrated anti-inflammatory effects through MAPK pathway modulation and improved OA-associated functional and structural changes. The chemical characterization of CMXP provides a basis for further investigation of its potential application in OA.

## 1. Introduction

Osteoarthritis (OA) is a prevalent condition that is influenced by factors such as age, sex, obesity, and joint trauma. OA is primarily characterized by cartilage damage resulting from biomechanical and biochemical changes within the joint. OA manifests clinically as joint pain, stiffness, and swelling. Radiographic examination is essential for diagnosis and reveals bony alterations such as osteophyte formation, cysts, subchondral sclerosis, and joint space narrowing [1].

Currently, OA management primarily relies on physical therapy, nonsteroidal anti-inflammatory drugs (NSAIDs), analgesics, and intraarticular injections of glucocorticoids or hyaluronic acid [2]. However, these approaches fail to adequately address the underlying pathological mechanisms of OA, highlighting the need for alternative therapeutic strategies targeting key inflammatory and degenerative pathways.

Macrophages play pivotal roles in both innate immunity and OA pathogenesis. Their activation can be triggered by various stimuli, including lipopolysaccharide (LPS), tumor necrosis factor (TNF-α), and interferon-gamma (IFN-γ). Activated macrophages secrete pro-inflammatory cytokines that contribute to cartilage degradation and bone loss [3,4]. This process establishes a detrimental cycle in which chondrocytes further release cytokines and proteolytic enzymes, thereby promoting synovial inflammation and exacerbating cartilage degradation. Increasing evidence indicates that modulation of inflammatory mediators—such as nitric oxide (NO), tumor necrosis factor-α (TNF-α), and interleukin-1β (IL-1β)—as well as intracellular signaling pathways, including MAPKs, plays a critical role in attenuating OA progression [5,6]. Notably, modulation of macrophage phenotypes has been suggested as a potential strategy to slow OA progression [7].

*Centipeda minima* (L.) A. Braun & Asch. is a botanical source rich in sesquiterpene lactones, including brevilin A, arnicolides C and D, and microhelenin C, which have demonstrated notable anti-inflammatory and analgesic properties. Phytochemical investigations have further identified flavonoids, essential oils, sterols, and phenolic compounds as additional bioactive constituents [8]. Several major sesquiterpene lactones have been reported to suppress inflammatory mediators and signaling pathways in macrophage-based models, supporting their potential relevance in inflammatory conditions [5]. While *C. minima* extracts have demonstrated anti-inflammatory effects in various preclinical models, their relevance to OA, particularly with respect to inflammatory and OA-associated degenerative pathways and MAPK signaling, remains insufficiently characterized.

Therefore, this study investigated the anti-inflammatory potential of chemically characterized *C. minima* extract (CMXP) using an LPS-stimulated macrophage inflammatory model and an MIA-induced osteoarthritis rat model. Specifically, we evaluated the inhibitory effects of four sesquiterpene lactones and CMXP on nitric oxide (NO) production in macrophages, followed by mechanistic studies focusing on MAPK signaling and inflammatory mediators. These in vitro findings were further evaluated in a monosodium iodoacetate (MIA)-induced OA rat model. Collectively, this study aims to provide a chemically characterized basis for evaluating CMXP in relation to its potential relevance in biopharmaceutical applications for OA management.

## 2. Results

### 2.1. Effects of Active Compounds of CMXP on Cell Viability and NO Levels in RAW264.7 Cells

**Figure 1:**
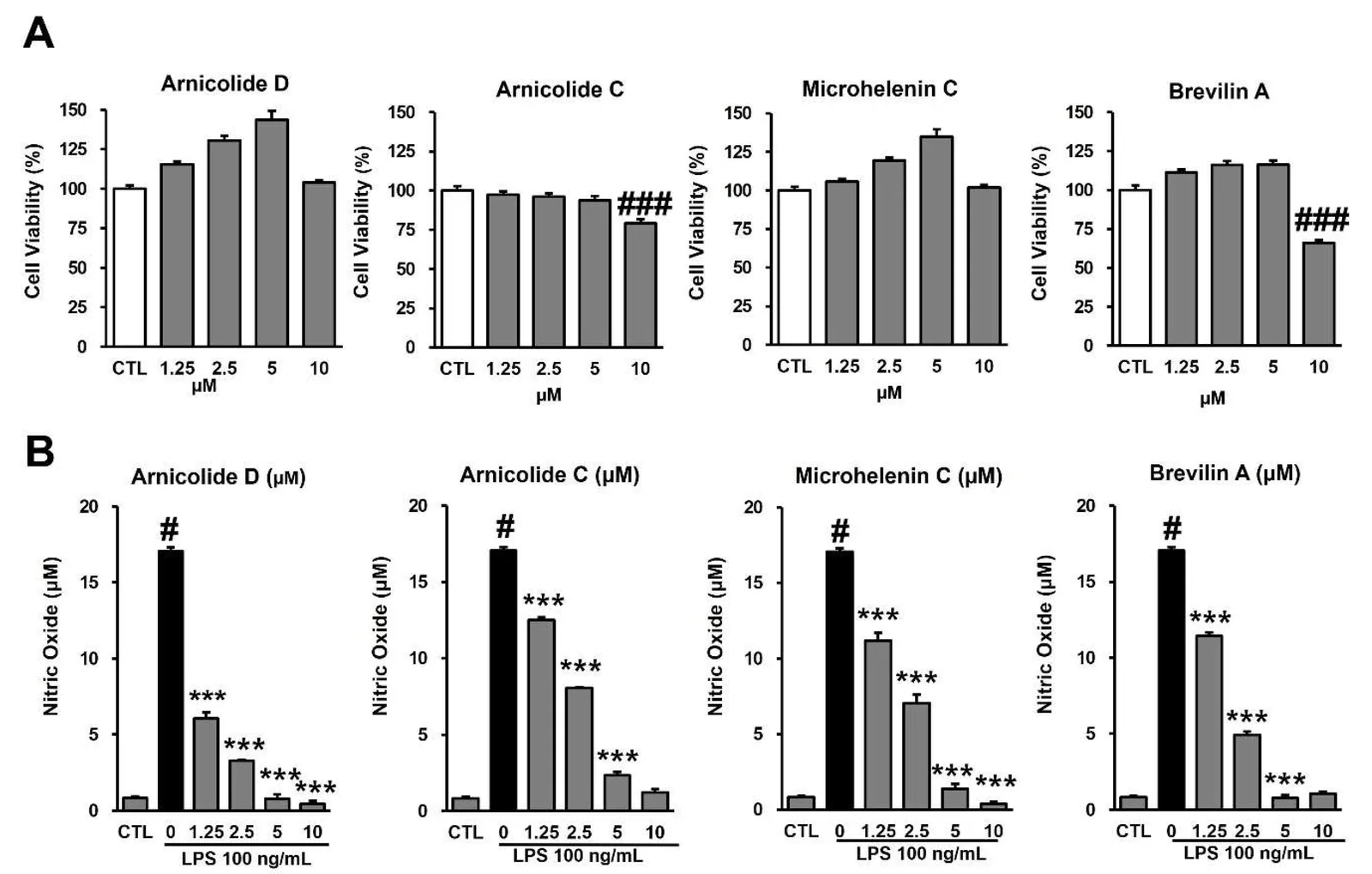
Effects of active compounds of CMXP on cytotoxicity and nitric oxide production in RAW264.7 cells. RAW264.7 cells were treated with the indicated concentrations of the samples for 24 h. Following treatment, cell viability (**A**) was assessed using the EZ-Cytox assay. For the NO assay (**B**), the cells were treated with various doses of the samples for 2 h and then induced with LPS. The results are reported as percentages relative to the control. Data are presented as mean ± SD of triplicate experiments. ^###^ *p* < 0.05 compared with the untreated group; ^#^ *p* < 0.05 compared with the untreated group; *** *p* < 0.05 compared with the LPS-treated group.

The effect of the active compounds from CMXP on cell viability was evaluated using the EZ-Cytox assay. As shown in Figure 1, the viability of cells treated with arnicolide D and microhelenin C increased in a dose-dependent manner, except at the highest concentration of 5 μM. No cytotoxicity was observed at 5 μM for either compound. In contrast, treatment with 10 μM of arnicolide C or brevilin A decreased viability to 78.95 ± 2.56 and 65.97 ± 1.78, respectively. Nitric oxide (NO) production was assessed using the Griess reagent. All four compounds inhibited LPS-induced nitrite oxide production in a concentration-dependent manner. However, because arnicolide C and brevilin A reduced cell viability at 10 μM, the NO-inhibitory effects observed at this concentration should be interpreted with caution.

### 2.2. Effect of CMXP on Macrophage Viability and NO Levels

**Figure 2:**
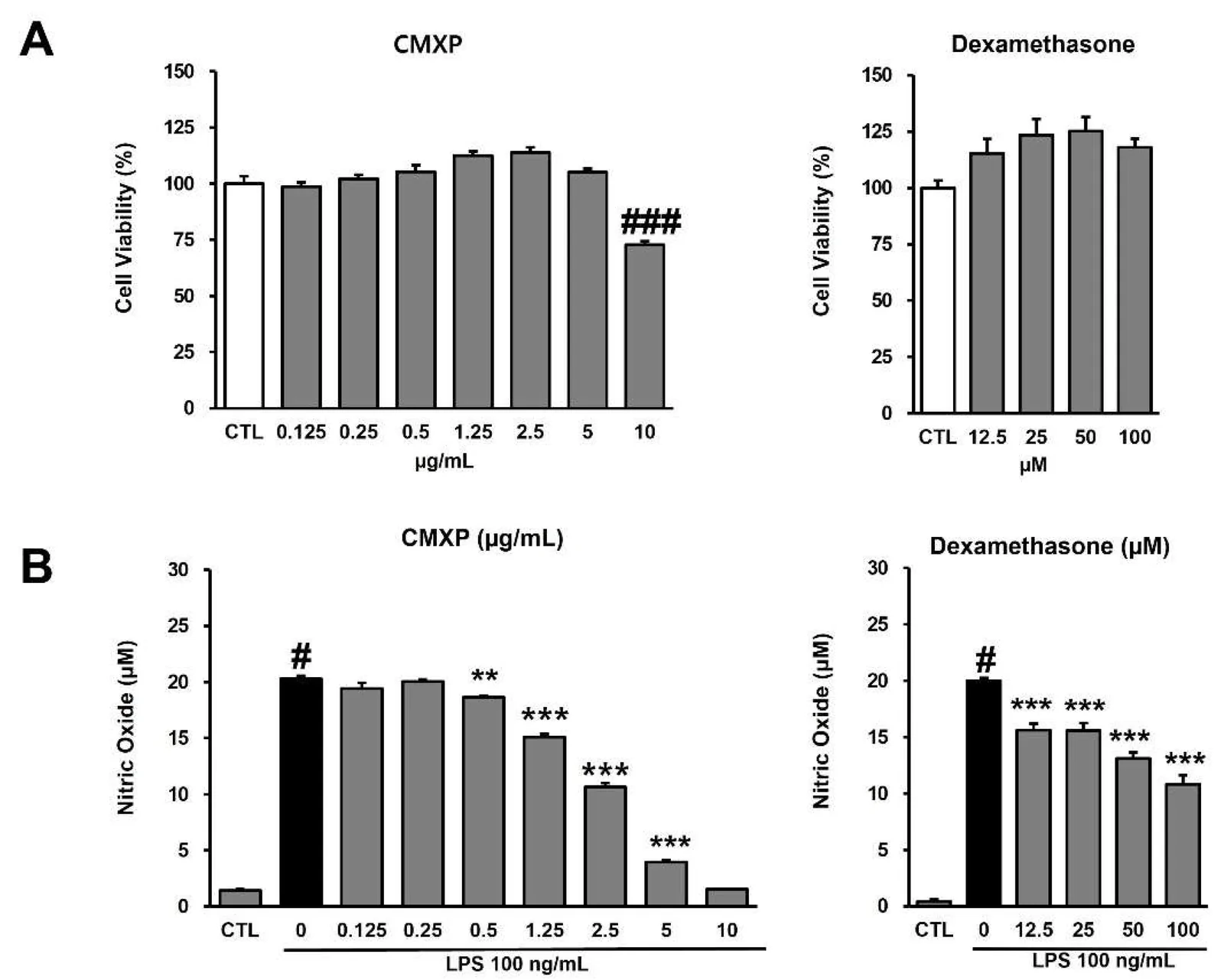
Effects of CMXP on cytotoxicity and nitric oxide production in LPS-activated RAW264.7 cells. RAW264.7 cells were treated with the indicated concentrations of the samples for 24 h. Following treatment, cell viability (**A**) was assessed using the EZ-Cytox assay. For the NO assay (**B**), the cells were treated with various doses of the samples for 2 h and then induced with LPS. Dexamethasone was used as a positive control. The results are reported as percentages relative to the control. Data are presented as mean ± SD of triplicate experiments. ^###^ *p* < 0.05 compared with the untreated group; ^#^ *p* < 0.05 compared with the untreated group; ** *p* < 0.05 and *** *p* < 0.001 compared with the LPS-treated group.

Based on the results shown in Figure 2, the effects of CMXP were further evaluated by assessing cytotoxicity (Figure 2A) and NO production (Figure 2B). RAW264.7 cells were treated with CMXP at concentrations ranging from 0.125 μg/mL to 10 μg/mL. CMXP showed no cytotoxicity up to 5 μg/mL, whereas reduced viability was observed at 10 μg/mL. Because CMXP reduced cell viability at 10 μg/mL, the NO-inhibitory effect observed at this concentration was interpreted with caution. CMXP significantly decreased NO production in a dose-dependent manner at non-cytotoxic concentrations. At 2.5 μg/mL, NO levels were reduced to 10.67 ± 0.33 µM compared to the control. Dexamethasone, used as a positive control, did not affect cell viability and exhibited a dose-dependent inhibitory effect on NO production.

### 2.3. Effects of CMXP on MAPK Signaling and Inflammatory Mediators in LPS-Induced RAW264.7 Cells

**Figure 3:**
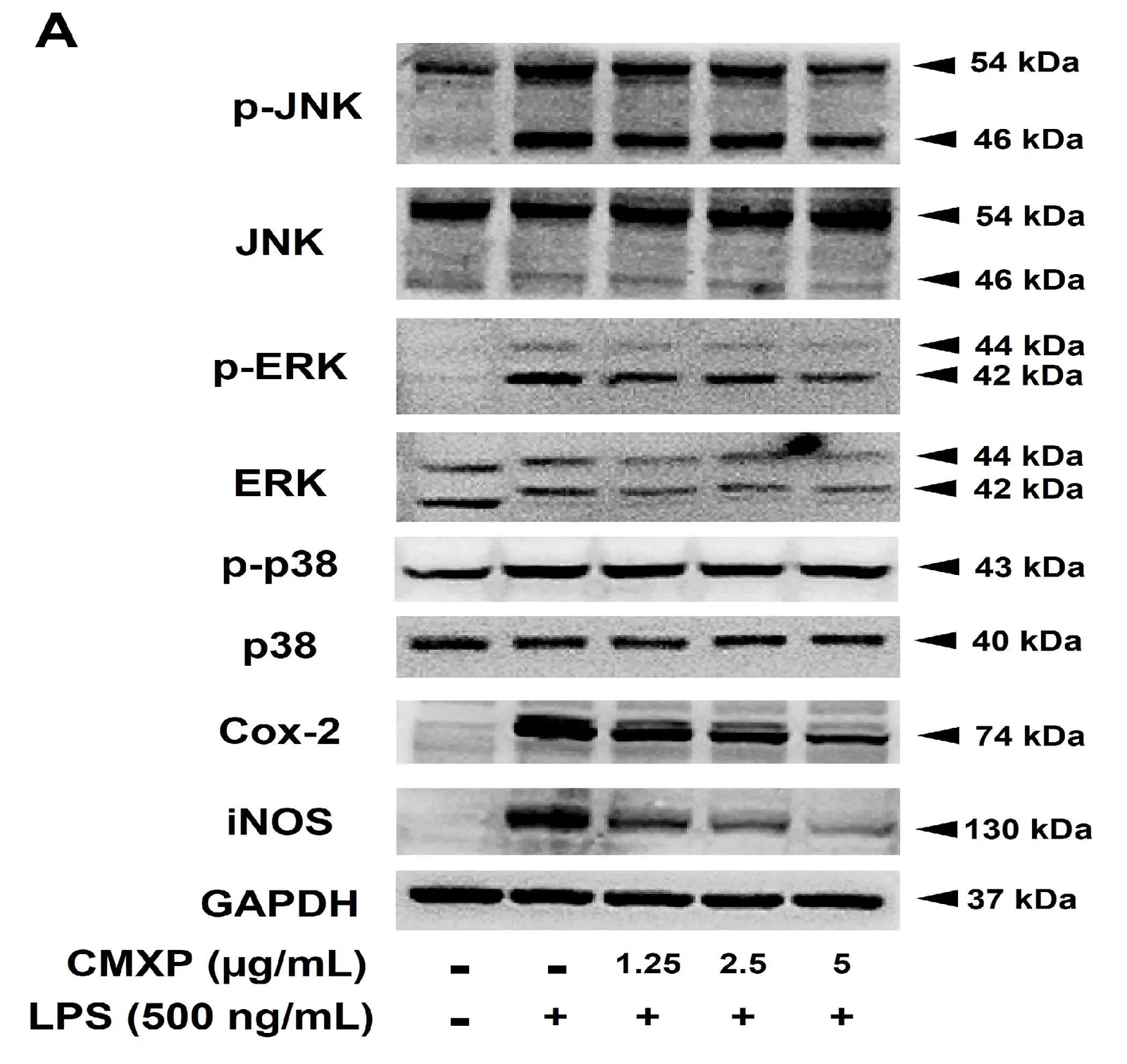

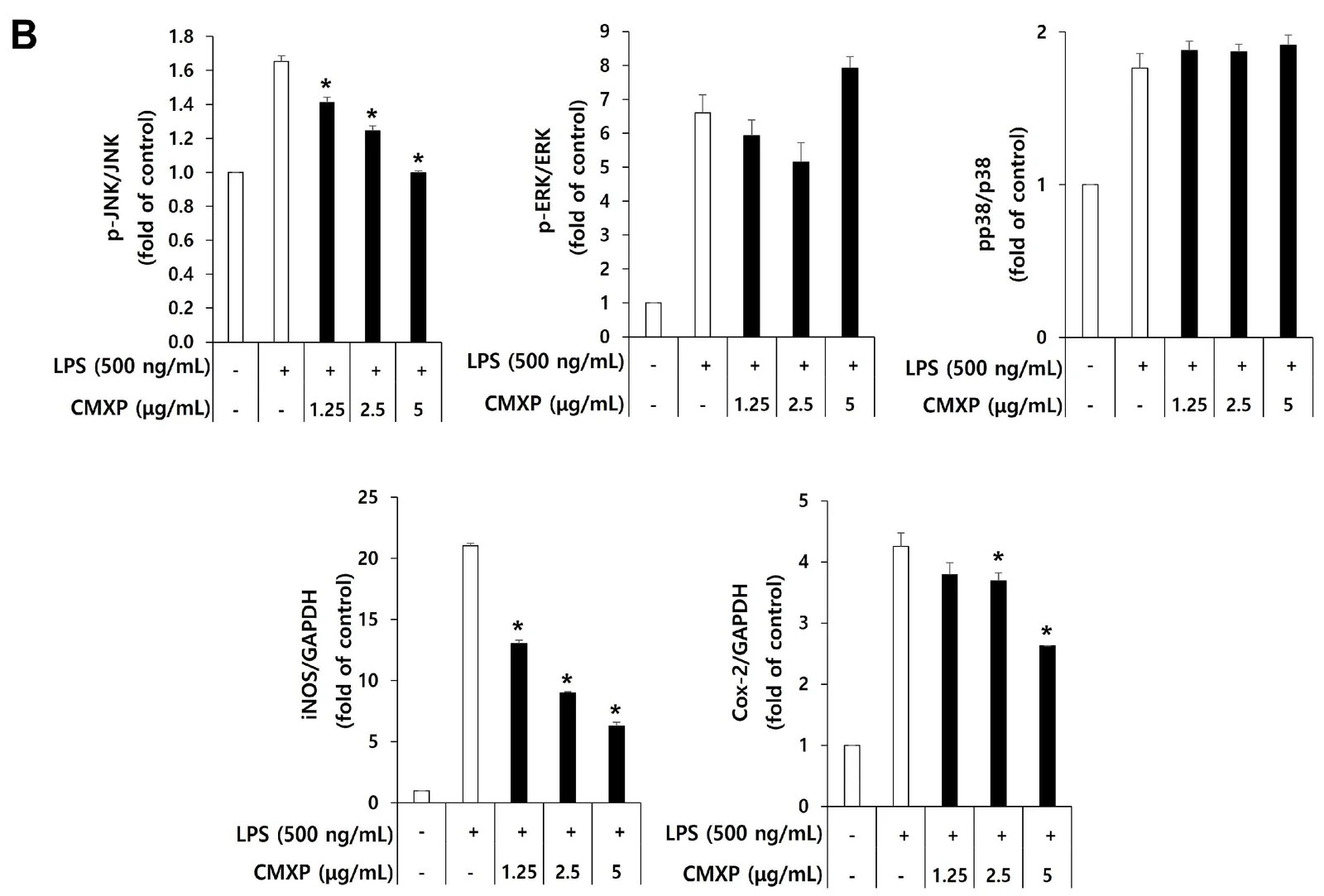
Effects of CMXP on MAPK signaling and inflammatory mediators in LPS-induced RAW264.7 cells. RAW264.7 cells were treated with CMXP for 2 h, followed by treatment with LPS. Total cellular extracts were analyzed with the indicated antibodies using Western blotting (**A**). The protein bands were densitometrically analyzed using the ImageJ software (version 1.51j8; National Institutes of Health, Bethesda, MD, USA) and expressed as fold changes relative to the control, which was set to 1 (**B**). Data are presented as mean ± standard deviation (SD) of triplicate experiments. * *p* < 0.05 compared with the LPS-treated group.

As illustrated in Figure 3, CMXP treatment significantly reduced the phosphorylation of c-Jun N-terminal kinase (JNK) in LPS-stimulated macrophages in a concentration-dependent manner. In contrast, no significant changes were observed in the phosphorylation levels of the p38 protein.

CMXP also reduced extracellular signal-regulated kinase (ERK) phosphorylation up to 2.5 μg/mL, although the inhibitory effect was less pronounced than that observed for JNK. Furthermore, CMXP significantly reduced the levels of cyclooxygenase-2 (COX-2) and inducible NO synthase (iNOS).

### 2.4. Effects of CMXP on Hind Limb Weight-Bearing in OA-Induced Rat Models

**Figure 4:**
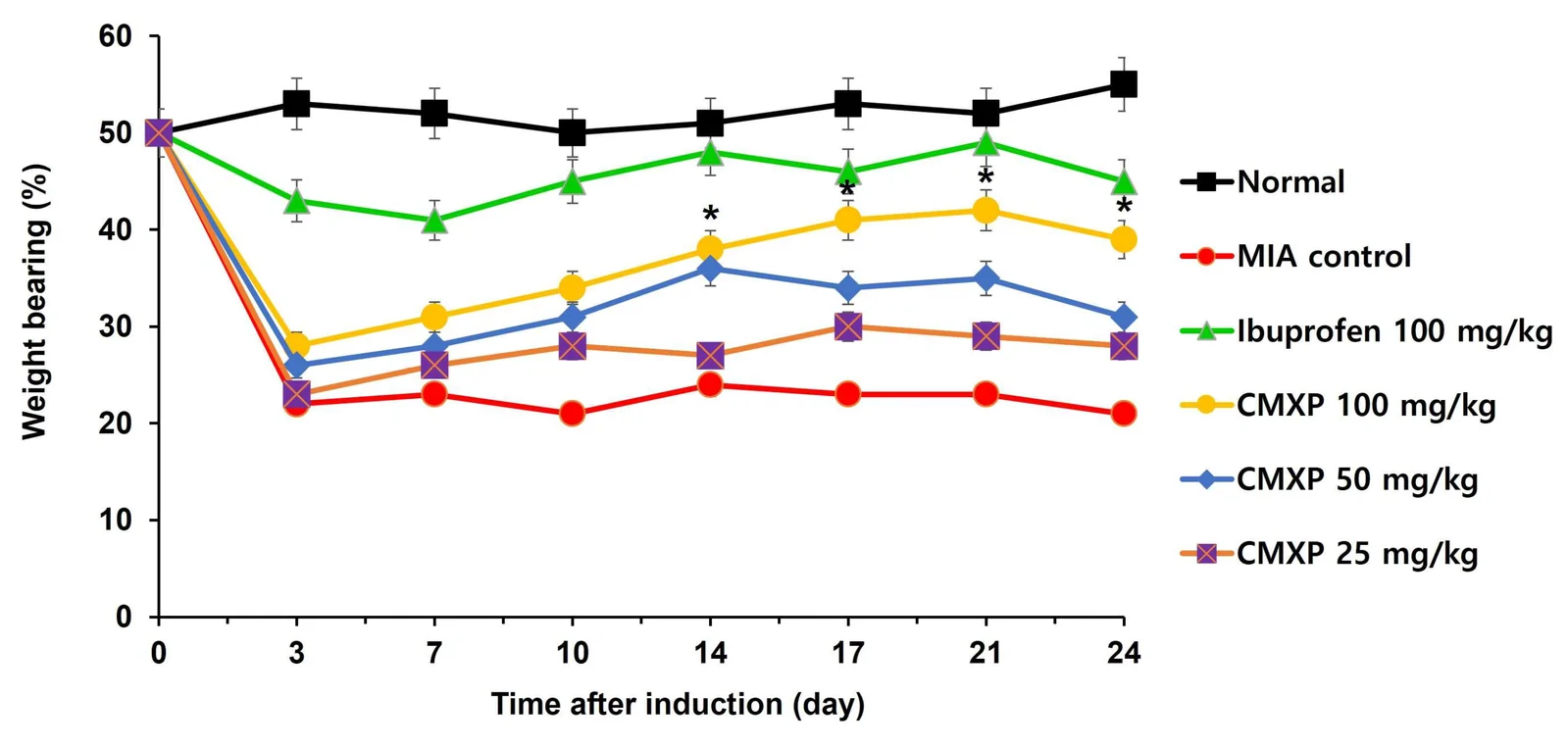
Effect of CMXP on hind limb weight-bearing in OA-induced rat models. MIA was injected once into the right knee joint at 50 mg/mL in a volume of 50 μL, corresponding to 2.5 mg MIA per joint. Hind limb weight-bearing was assessed using an Incapacitance Meter Tester on days 0, 3, 7, 10, 14, 17, 21, and 24. The values were calculated as the average over a 10-s period. The hind limb weight-bearing load across all groups was evaluated as a potential indicator of pain. * *p* < 0.05 compared to the control group.

As presented in Figure 4, CMXP was orally administered at doses of 25, 50, or 100 mg/kg or a vehicle to evaluate the effects of CMXP in the MIA-induced OA model. CMXP-treated groups showed a dose-dependent improvement in hind limb weight-bearing compared with the OA control group (induced with monoiodoacetate, MIA). Although a reduction in hind limb weight-bearing was observed in all OA groups at day 3 post-induction, the CMXP-treated groups gradually recovered in a dose-dependent manner. In contrast, the normal (non-OA) group maintained a stable hind limb weight distribution throughout the experiment. Ibuprofen was used as a positive control.

### 2.5. Effects of CMXP on Cartilage Degradation and Inflammatory Markers in OA Rats

**Figure 5:**
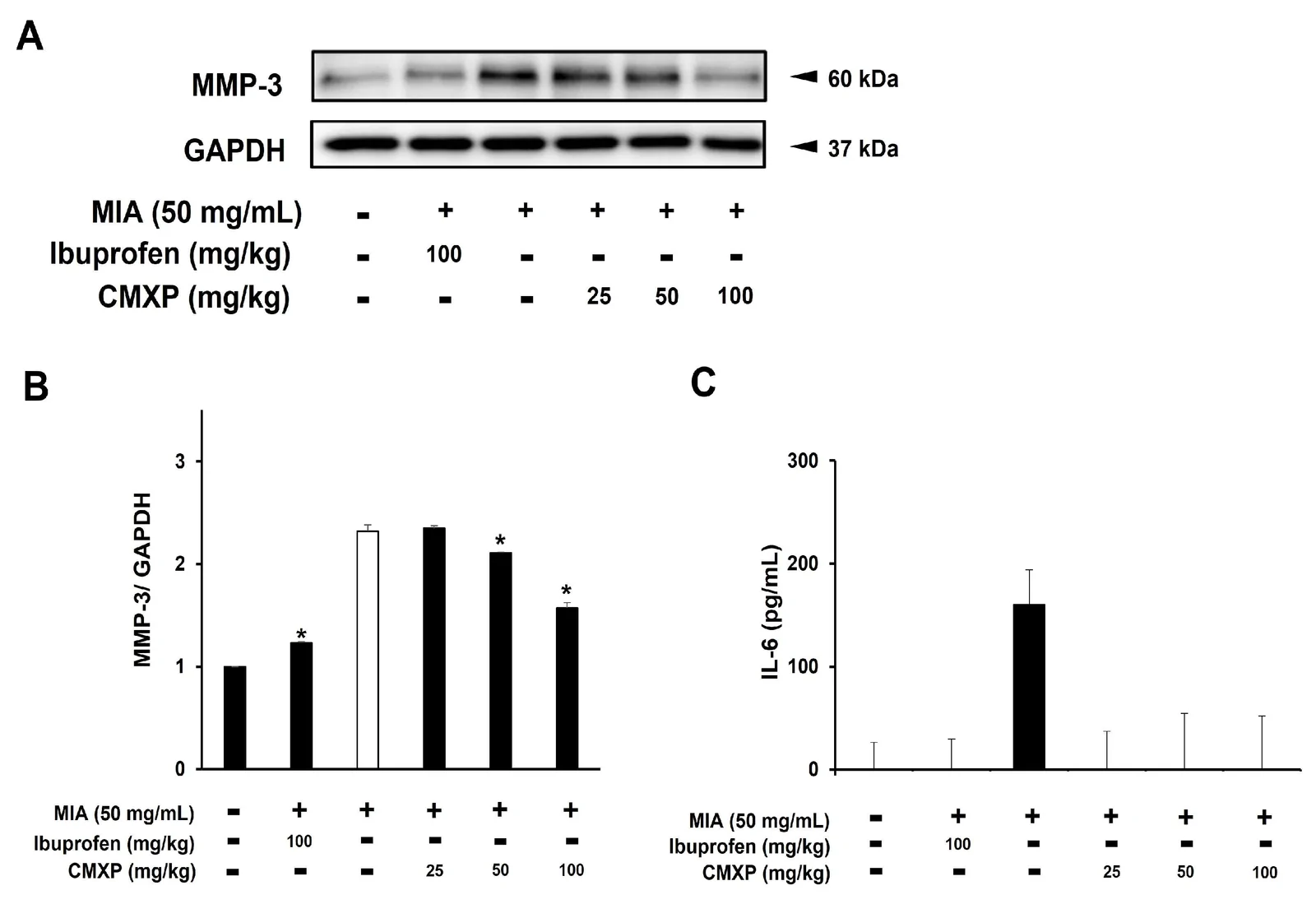
The effects of CMXP on cartilage degradation and serum IL-6 levels in OA-induced models. Protein expression of MMP-3 in knee cartilage tissue was analyzed by Western blot (**A**), and representative Western blot images are shown. The protein expression was quantified by densitometry (**B**), and the data are presented as mean ± SD (*n* = 3 rats per group). The concentration of IL-6 in the rat serum was assessed using ELISA at different doses of CMXP (**C**). The experimental groups were as follows: the normal group without MIA induction; MIA control, the MIA-induced group; Ibuprofen, the positive control group; and CMXP, the MIA-induced group treated with CMXP. * *p* < 0.05 compared with the MIA control group.

Matrix metalloproteinases (MMPs) are involved in extracellular matrix degradation and contribute to cartilage breakdown and OA progression. To evaluate the effect of CMXP, protein expression of MMP-3 was analyzed in cartilage tissue (Figure 5A,B). MIA induction increased MMP-3 expression compared with the normal group, whereas CMXP treatment reduced MMP-3 expression compared with the OA control group. In addition, CMXP administration decreased serum levels of IL-6 (Figure 5C), which were elevated in the OA control group. Both CMXP and ibuprofen treatments suppressed IL-6 levels, indicating anti-inflammatory effects.

### 2.6. Effects of CMXP on OA-Induced Morphological Changes in an In Vivo Model: A Micro-CT Analysis

**Figure 6:**
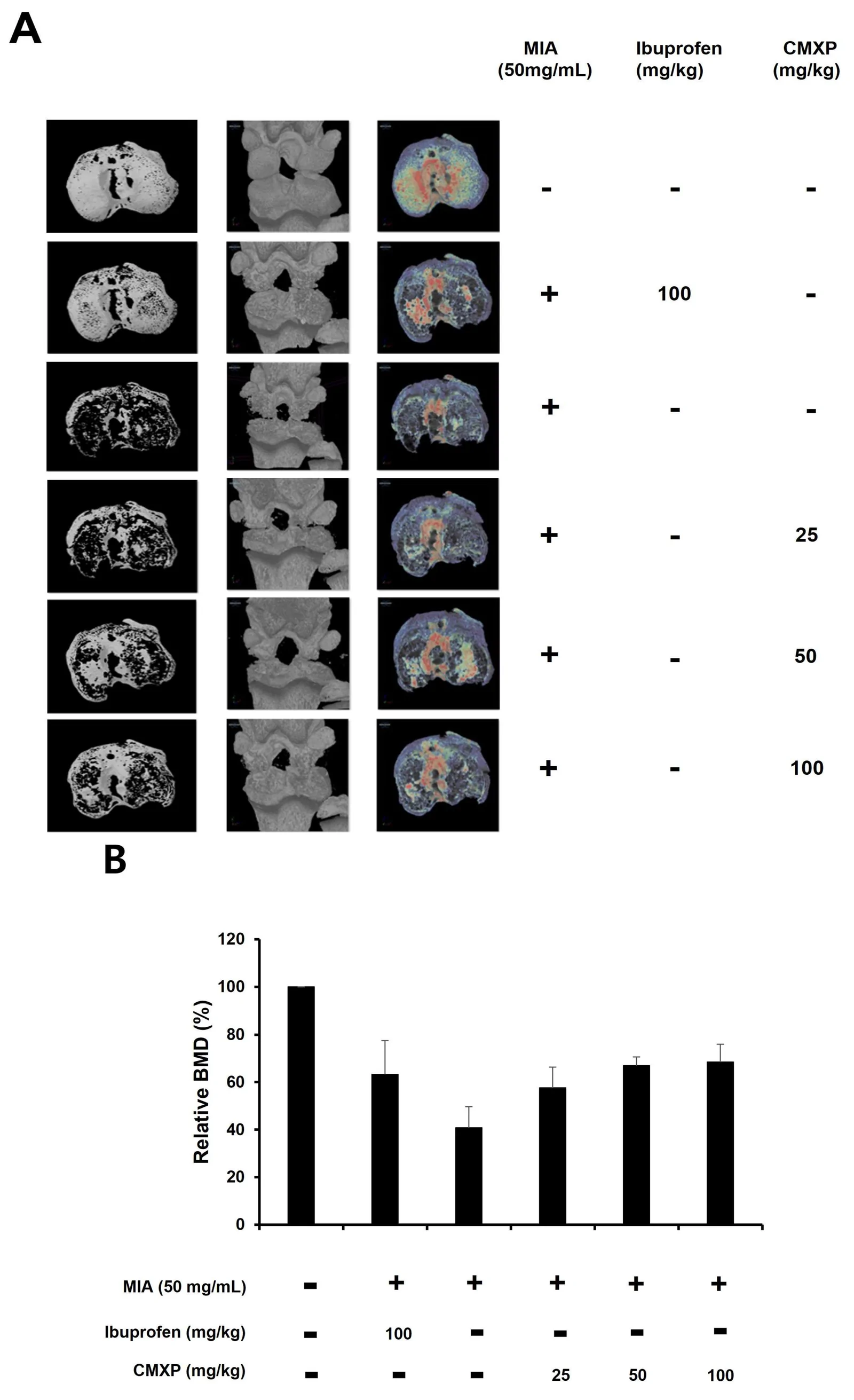
Effects of CMXP on subchondral bone changes in MIA-induced OA rats. The right hind limb was excised and fixed in formaldehyde, and micro-CT imaging was performed to assess the bone structural changes in rats from each experimental group (**A**). From left to right, the images show a top-down view of the joint, a sagittal view of the knee joint, and a 3D reconstruction with color mapping. The color mapping was used to visualize bone density distribution, whereas quantitative BMD values are presented in (**B**). Relative bone mineral density (BMD) was quantified within the subchondral bone of the tibial plateau. BMD values were normalized to the mean value of the normal group and expressed as relative BMD (%) (**B**). Data are presented as mean ± SD (*n* = 3 rats per group).

Micro-computed tomography (micro-CT) analysis was performed to evaluate bone structural changes (Figure 6). In the OA group, bone mineral density (BMD) and microstructure were reduced compared to the normal group. CMXP treatment attenuated the MIA-induced reduction in relative BMD, with higher mean values observed at increasing doses of CMXP. The ibuprofen-treated group also showed a higher relative BMD value than the MIA control group.

## 3. Discussion

In the present study, we demonstrated that a chemically characterized extract of *C. minima* (CMXP) and its constituent sesquiterpene lactones exert anti-inflammatory effects and modulate OA-associated degenerative changes in vitro and in vivo. CMXP attenuated LPS-induced inflammatory responses in RAW264.7 macrophages by suppressing iNOS and COX-2 expression and reducing nitric oxide production. Given that OA is characterized by chronic low-grade inflammation, progressive cartilage degradation, and subchondral bone remodeling [9], these findings indicate that CMXP modulates key inflammatory pathways associated with OA pathogenesis.

The in vivo results further support these observations. In the MIA-induced OA rat model, CMXP administration improved hind limb weight-bearing function and preserved structural features of the joint. CMXP suppressed IL-6 and MMP-3 expression [10] and mitigated the loss of subchondral bone mineral density [11], suggesting effects on both inflammatory and structural aspects of OA. Compared to conventional NSAIDs, which primarily target symptomatic relief, these results suggest that CMXP may influence multiple pathological processes associated with disease progression [9].

A notable aspect of this study is the comparison between the NO-inhibitory activity of the isolated sesquiterpene lactones and that of CMXP. The isolated compounds were evaluated at micromolar concentrations, whereas CMXP was tested as a complex extract at concentrations expressed in μg/mL. According to the quantitative analysis of CMXP, 2.5 μg/mL CMXP, a concentration that significantly inhibited NO production [12] without marked cytotoxicity, nominally contained approximately 0.584 μg/mL brevilin A and 0.177 μg/mL arnicolide D. Similarly, oral CMXP doses of 25, 50, and 100 mg/kg corresponded to approximately 5.84, 11.68, and 23.36 mg/kg of brevilin A and 1.77, 3.55, and 7.09 mg/kg of arnicolide D, respectively. However, these nominal constituent amounts cannot be directly equated with the micromolar concentrations used in the isolated-compound experiments because CMXP contains multiple constituents and the absorption, metabolism, systemic exposure, and tissue distribution of individual components were not determined. Therefore, the present data do not permit direct potency comparisons or conclusions regarding additive or synergistic interactions among CMXP constituents.

Mechanistically, CMXP modulates signaling pathways relevant to OA by inhibiting MAPK activation, particularly ERK and JNK [13], and reducing downstream inflammatory mediators [14]. In addition, CMXP decreased cytokine production [15] and MMP expression [10] while preserving bone mineral density and improving joint function in vivo. Collectively, these findings support the anti-inflammatory effects of CMXP and its ability to modulate OA-associated cartilage degradation markers and subchondral bone changes.

Despite these findings, several limitations should be considered. Because the isolated compounds and CMXP were tested using different concentration units and exposure conditions, their relative potencies cannot be directly compared in the present study. In addition, the pharmacokinetic and bioavailability profiles of CMXP were not investigated, which limits the interpretation of its in vivo efficacy. Furthermore, the treatment duration may not fully reflect long-term safety or sustained effects.

Future studies should further characterize the interactions among CMXP constituents using in vitro combination experiments with concentration–response matrices, followed by quantitative interaction analyses such as combination index or response-surface analysis. Network pharmacology and metabolomic profiling may also help identify shared molecular targets and pathway-level interactions among CMXP constituents. In addition, LC-MS/MS-based pharmacokinetic studies should be performed to determine the absorption, systemic exposure, metabolism, and tissue distribution of major sesquiterpene lactones following oral administration of CMXP. Validation in additional OA models and extended study durations will also be necessary to better understand the translational relevance of CMXP.

Taken together, the present findings demonstrate that CMXP attenuates inflammatory responses through modulation of MAPK signaling while improving functional and structural outcomes in an experimental OA model. These results support the further investigation of CMXP as a chemically characterized botanical extract for osteoarthritis.

## 4. Materials and Methods

### 4.1. Plant Material

*Centipeda minima* (L.) A. Braun & Asch. was purchased from Natural-herb Co. (Goesan, Republic of Korea). A voucher specimen (CM-2019-001) was deposited in the herbarium at Kyungsung University.

### 4.2. Preparation of CMXP

CMXP was prepared as previously described [16,17]. The aerial parts of *C. minima* (20 kg, chopped) were extracted with 10 volumes of 30% ethanol under continuous stirring for 24 h. The crude extract was then passed through 5 kg of HP-20 resin. After thorough washing with water to remove unbound materials, the adsorbed fractions were eluted using 10 L of 50% ethanol. Medium-chain triglyceride (MCT) oil was added to the eluate, and the mixture was stirred, followed by evaporation of the organic solvent using a rotary evaporator (N-1300E; Tokyo Rikakikai Co., Ltd., Tokyo, Japan). After phase separation, the upper layer was collected and dried to obtain CMXP.

### 4.3. Chemical Characterization of CMXP

**Figure 7:**
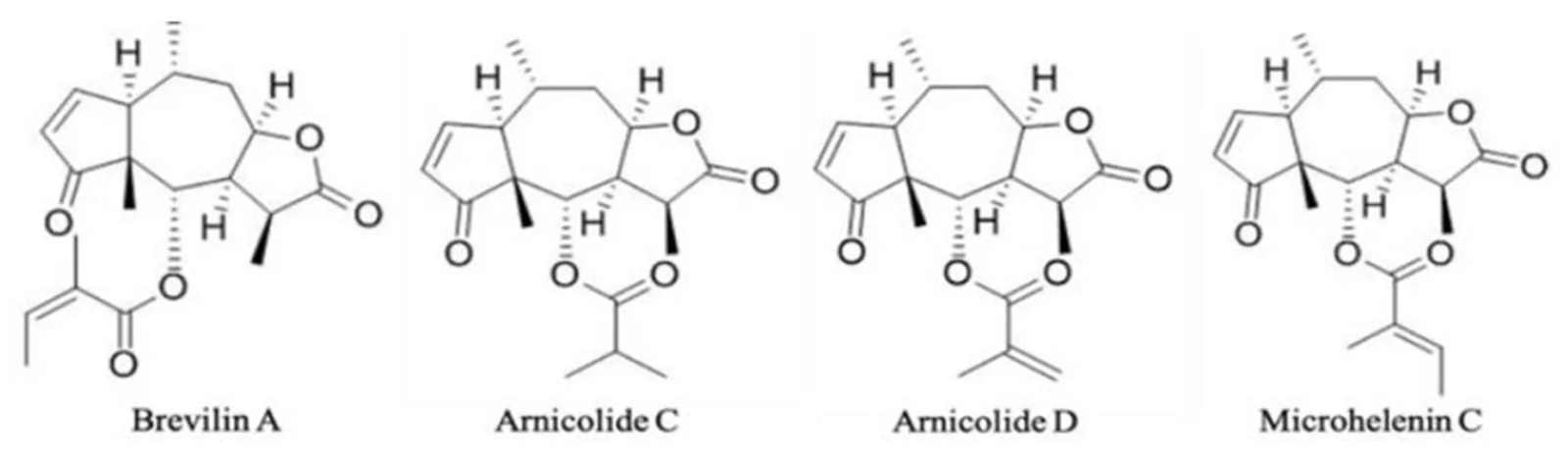
Chemical structures of brevilin A, arnicolid C, arnicolid D, and microhelenin C.

CMXP was chemically characterized based on selected sesquiterpene lactone constituents, including arnicolide C, arnicolide D, micro-helenin C, and brevilin A (Figure 7). The marker compounds (arnicolide C, arnicolide D, micro-helenin C, and brevilin A) were determined by high-performance liquid chromatography (HPLC) using authentic reference standards (Figure S1). The CMXP batch used in this study (Batch No. CMXP-22001) was obtained as a semisolid, containing 233.6 mg/g of brevilin A and 70.9 mg/g of arnicolide D.

### 4.4. Cell Culture and Cytotoxicity Assay

The murine macrophage cell line, RAW264.7, was cultured in DMEM (Corning, Manassas, VA, USA) supplemented with 10% FBS (Atlas Biologicals, Fort Collins, CO, USA), penicillin G (100 U/mL), and streptomycin (100 μg/mL) (Gibco BRL, Carlsbad, MD, USA) at 37 °C in a 5% CO_2_ humidified atmosphere. Cell viability was evaluated using the EZ-Cytox assay (DoGenBio, Seoul, Republic of Korea) as previously described [18].

### 4.5. Nitrite Assay

Macrophages were seeded into a 96-well plate at a density of 5 × 10^4^ cells/well. Cells were pretreated with the indicated concentrations. After 2 h, the cells were treated with 500 ng/mL LPS (Sigma-Aldrich, St. Louis, MO, USA) for 22 h. Each supernatant was transferred to a new 96-well plate and mixed with an equal volume of Griess reagent [19]. Absorbance was measured at 550 nm using a microplate reader (SPARK 10M; Tecan, Männedorf, Switzerland).

### 4.6. Western Blotting

RAW264.7 cells were treated with CMXP for 2 h, followed by LPS treatment for 22 h, and then the cells were harvested. This analysis was performed as previously described [20].

### 4.7. MIA-Induced OA Rat Model

Male Sprague Dawley rats, 5 weeks of age and weighing approximately 180 g at purchase, were obtained from DBL (Eumseong, Republic of Korea). The animals were housed under controlled conditions at 22 ± 2 °C and 55 ± 10% relative humidity under a 12 h light/dark cycle (lights on from 07:00 to 19:00), with free access to food and water. The rats were acclimatized for at least 7 days before the experiment. Prior to MIA administration, the animals were assigned to six experimental groups (*n* = 10 per group) so that body weights were evenly distributed among groups: the Normal group, the MIA control group, the Ibuprofen group, and the CMXP 25, CMXP 50, and CMXP 100 groups. Anesthesia was induced using 2% isoflurane in an O_2_ mixture. MIA (Sigma-Aldrich, St. Louis, MO, USA) [21] was dissolved in 0.9% saline to achieve a final concentration of 50 mg/mL, and 50 μL of solution was injected once into the right knee using a 26-gauge, 0.5-inch needle, corresponding to 2.5 mg MIA per joint. Oral administration was conducted for 24 d, beginning with the induction of OA with MIA. This study was performed in accordance with the ethical standards of the Institutional Animal Care and Use Committee of Gachon University (Approval No.: Gu1-2022-IA0014-00).

### 4.8. Drug Administration

CMXP was administered orally once daily for 24 consecutive days, starting on the day of OA induction. CMXP was suspended in 0.5% carboxymethylcellulose (CMC) solution for oral administration. The MIA control group received the same vehicle without CMXP. Three dosage levels of CMXP (25, 50, and 100 mg/kg) were selected based on a previous study reporting no treatment-related toxicity following repeated oral administration of CMXP for 1 month at doses up to 400 mg/kg [6]. CMXP 25, CMXP 50, and CMXP 100 groups received CMXP at doses of 25, 50, and 100 mg/kg, respectively, corresponding to combined amounts of 7.61, 15.23, and 30.46 mg/kg of brevilin A and arnicolide D, respectively. The dosing volume was standardized at 10 mL/kg body weight for all groups. For the positive control, ibuprofen was incorporated into the feed to reach a final dose of 100 mg/kg, whereas the normal control group was provided with standard feed.

### 4.9. Hind Limb Weight-Bearing Test

Weight-bearing distribution between hind limbs was measured using an Incapacitance Meter Tester 600 (IITC Life Science Inc., Woodland Hills, CA, USA) at days 0, 3, 7, 10, 14, 17, 21, and 24 post-induction. This measurement was averaged over a 10 s interval. The weight-bearing ratio (%) was calculated as follows: (weight on right hind limb/[weight on left hind limb + weight on right hind limb]) × 100.

### 4.10. Serum IL-6 and Cartilage Protein Analysis

On day 24 after MIA induction, blood samples were collected and allowed to clot at room temperature for 30 min, followed by centrifugation at 3000 rpm for 10 min to obtain serum. IL-6 levels were quantified using an ELISA assay according to the manufacturer’s instructions [22]. Total protein was extracted from the OA knee cartilage tissue using RIPA buffer containing a complete Mini EDTA-free Protease Inhibitor Cocktail. Protein expression levels of MMP-3 and GAPDH were analyzed by Western blotting.

### 4.11. Micro-Computed Tomography Analysis

At day 24 post-induction, the right hind limbs were excised and fixed in formaldehyde. Structural changes in the knee joint were evaluated by a micro-computed tomography (micro-CT) system (SkyScan 1276; Bruker, Kontich, Belgium). The acquisition parameters were rigorously standardized across all samples to ensure consistency: source voltage of 70 kV, source current of 300 μA, an aluminum filter of 0.5 mm, and a nominal isotropic voxel size of 18 μm. For calibration of bone mineral density, phantom rods containing known concentrations of dipotassium phosphate (K_2_HPO_4_) were scanned under identical acquisition conditions. The two-dimensional images were reconstructed using NRecon software (v1.7.4; Bruker) with a smoothing factor of 2, ring artifact reduction of 5, and beam hardening correction of 30%. For quantitative analysis, the region of interest (ROI) was defined within the subchondral bone of the tibial plateau, spanning 100 consecutive slices (approximately 1.8 mm), with the same anatomical reference point applied to all samples. A global threshold (gray values, 60–255) was applied to segment the mineralized bone tissue from the background. Absolute volumetric bone mineral density (vBMD) values obtained after calibration were subsequently normalized to the mean value of the normal group and expressed as relative BMD (%).

### 4.12. Statistical Analysis

Statistical analyses were performed by GraphPad Prism software (version 5.0; GraphPad Software Inc., La Jolla, CA, USA). All data are presented as the mean ± standard deviation (SD). Comparisons among multiple groups were analyzed using one-way ANOVA followed by Tukey’s multiple-comparison test. Hind limb weight-bearing data were analyzed using two-way repeated-measures ANOVA followed by Tukey’s multiple-comparison test. A value of *p* < 0.05 was considered statistically significant.

## 5. Conclusions

This study demonstrates that CMXP exerts anti-inflammatory effects and improves OA-associated functional and structural outcomes in a rat model of osteoarthritis. CMXP suppressed inflammatory responses through inhibition of MAPK signaling and improved OA-associated structural changes. These findings support further investigation of CMXP as a chemically characterized botanical extract for osteoarthritis management.

## Data Availability

The data supporting the findings of this study are available from the corresponding authors upon reasonable request.

## Supplementary Materials

The following supporting information can be downloaded at https://www.mdpi.com/article/10.3390/molecules31203639/s1, Figure S1: High-performance liquid chromatography (HPLC) chromatograms of CMXP. Extraction and analysis were performed according to previously described methods [17]. The chromatogram was reproduced with permission under the Creative Attribution License (CC BY 4.0 license).

## Abbreviations

The following abbreviations are used in this manuscript:

CMXP	*Centipeda minima* extract
MIA	monosodium iodoacetate
OA	osteoarthritis
NO	nitric oxide
MAPK	mitogen-activated protein kinase
JNK	c-Jun N-terminal kinase
iNOS	inducible NO synthase
COX-2	cyclooxygenase-2
ERK	extracellular signal-regulated kinase
MMP	matrix metalloproteinase
LPS	lipopolysaccharide
TNF-α	tumor necrosis factor
IFN-γ	interferon-gamma
NSAIDs	nonsteroidal anti-inflammatory drugs
IL-1β	interleukin-1β
μ-CT	micro-computed tomography

## References

[1] Shin M.-R., Kim M., An H.Y., Choi H.-Y., Ham Y., Choi H., Roh S.-S. (2024). Effects of oral hyaluronic acid on monosodium iodoacetate-induced osteoarthritis in rats: Mechanistic insights and therapeutic implications. Appl. Biol. Chem..

[2] Kim M., Kim M.H., Kim J., Kang K., Lee J., Ghosh M., Son Y.-O. (2024). Comprehensive in vitro and in vivo investigations of the therapeutic potential of Jeju lava seawater salt in osteoarthritis. Appl. Biol. Chem..

[3] Kim J.-H., Lee R.M., Oh H.-B., Kim T.-Y., Rhim H., Choi Y.K., Kim J.-H., Oh S., Kim D.-G., Cho I.-H. (2024). Atypical formations of gintonin lysophosphatidic acids as new materials and their beneficial effects on degenerative diseases. J. Ginseng Res..

[4] Le M.H., Ahn Y.H., Lee H.-J., Kim Y.J. (2024). Stem-and-leaf of new hydroponically-cultured ginseng cultivar K-1: A sustainable and innovative resource of ginsenosides for anti-inflammatory agents. J. Ginseng Res..

[5] Huang S.-S., Chiu C.-S., Lin T.-H., Lee M.-M., Lee C.-Y., Chang S.-J., Hou W.-C., Huang G.-J., Deng J.-S. (2013). Antioxidant and anti-inflammatory activities of aqueous extract of *Centipeda minima*. J. Ethnopharmacol..

[6] Li S.-Y., Zhou Y.-L., He D.-H., Liu W., Fan X.-Z., Wang Q., Pan H.-F., Cheng Y.-X., Liu Y.-Q. (2020). *Centipeda minima* extract exerts antineuroinflammatory effects via the inhibition of NF-κB signaling pathway. Phytomedicine.

[7] Zhang H., Cai D., Bai X. (2020). Macrophages regulate the progression of osteoarthritis. Osteoarthr. Cartil..

[8] Liu J., Zheng W., He Y., Zhang W., Luo Z., Liu X., Jiang X., Meng F., Wu L. (2023). A Review of the Research Applications of *Centipeda minima*. Molecules.

[9] Kapoor M., Martel-Pelletier J., Lajeunesse D., Pelletier J.-P., Fahmi H. (2011). Role of proinflammatory cytokines in the pathophysiology of osteoarthritis. Nat. Rev. Rheumatol..

[10] Malemud C.J. (2006). Matrix metalloproteinases (MMPs) in health and disease: An overview. Front. Biosci.

[11] Hu H., Yang B., Li Y., Zhang S., Li Z. (2016). Blocking of the P2X7 receptor inhibits the activation of the MMP-13 and NF-κB pathways in the cartilage tissue of rats with osteoarthritis. Int. J. Mol. Med..

[12] Tanno O., Ota Y., Kitamura N., Katsube T., Inoue S. (2000). Nicotinamide increases biosynthesis of ceramides as well as other stratum corneum lipids to improve the epidermal permeability barrier. Br. J. Dermatol..

[13] Kaminska B. (2005). MAPK signalling pathways as molecular targets for anti-inflammatory therapy—From molecular mechanisms to therapeutic benefits. Biochim. Biophys. Acta.

[14] Cho J.Y., Baik K.U., Jung J.H., Park M.H. (2000). In vitro anti-inflammatory effects of cynaropicrin, a sesquiterpene lactone, from *Saussurea lappa*. Eur. J. Pharmacol..

[15] Guo Q., Jin Y., Chen X., Ye X., Shen X., Lin M., Zeng C., Zhou T., Zhang J. (2024). NF-κB in biology and targeted therapy: New insights and translational implications. Signal Transduct. Target. Ther..

[16] Kim B.H., Lee W.-Y., Trinh T.A., Pyo J.S., Lee S., Kim C.-E., Lee D.H., Park E.-S., Kang K.S. (2020). Hair growth effect of emulsion extracted brevilin A, a JAK3 inhibitor, from *Centipeda minima*. Processes.

[17] Kim B.H., Lee M.J., Lee W.-Y., Pyo J., Shin M.-S., Hwang G.S., Shin D., Kim C.E., Park E.-S., Kang K.S. (2021). Hair growth stimulation effect of *Centipeda minima* extract: Identification of active compounds and anagen-activating signaling pathways. Biomolecules.

[18] Tran N.K.S., Nguyen N.Q., Lee S., Kim S.H., Jeong D., Seo E., Park J.J., Cho J., Kang K.S. (2024). Anticancer effects of aloe-emodin from *Rheum undulatum* L. through activation of the p53 pathway in human prostate cancer cells. Appl. Biol. Chem..

[19] Kim K.M., Im A.-R., Shim K.-S., Seo C.-S., Lee Y., Lee J., Yoo J.S., Choi S., Chae S. (2024). Chikusetsusaponin IVa from *Dolichos lablab* Linne attenuates UVB-induced skin photoaging in mice by suppressing MAPK/AP-1 signaling. Appl. Biol. Chem..

[20] Truong V.-L., Bae Y.-J., Bang J.-H., Jeong W.-S. (2024). Combination of red ginseng and velvet antler extracts prevents skin damage by enhancing the antioxidant defense system and inhibiting MAPK/AP-1/NF-κB and caspase signaling pathways in UVB-irradiated HaCaT keratinocytes and SKH-1 hairless mice. J. Ginseng Res..

[21] Wu P., Su M.-X., Wang Y., Wang G.-C., Ye W.-C., Chung H.-Y., Li J., Jiang R.-W., Li Y.-L. (2012). Supercritical fluid extraction assisted isolation of sesquiterpene lactones with antiproliferative effects from *Centipeda minima*. Phytochemistry.

[22] Zhou K., Yu H., Duan H., Yang Y., Sheng W., Qiu Y., Wang W., Li B. (2025). Total saponins of Panax japonicus alleviate adjuvant-induced arthritis in rats by regulating macrophage polarization and intestinal microbiota. J. Ginseng Res..

