# Supplementary material for: A Chemically Characterized Extract of Centipeda minima Attenuates Inflammation in Macrophages and Alleviates Osteoarthritis Progression in Rats
Source: Molecules. 2026 Oct 12;31(20):3639. doi: 10.3390/molecules31203639 (PMC13707515; doi:10.3390/molecules31203639)
Supplement: Supplementary file 1 [file molecules-31-03639-s001.zip › molecules-4601377-supplementary.pdf]

Supplementary data

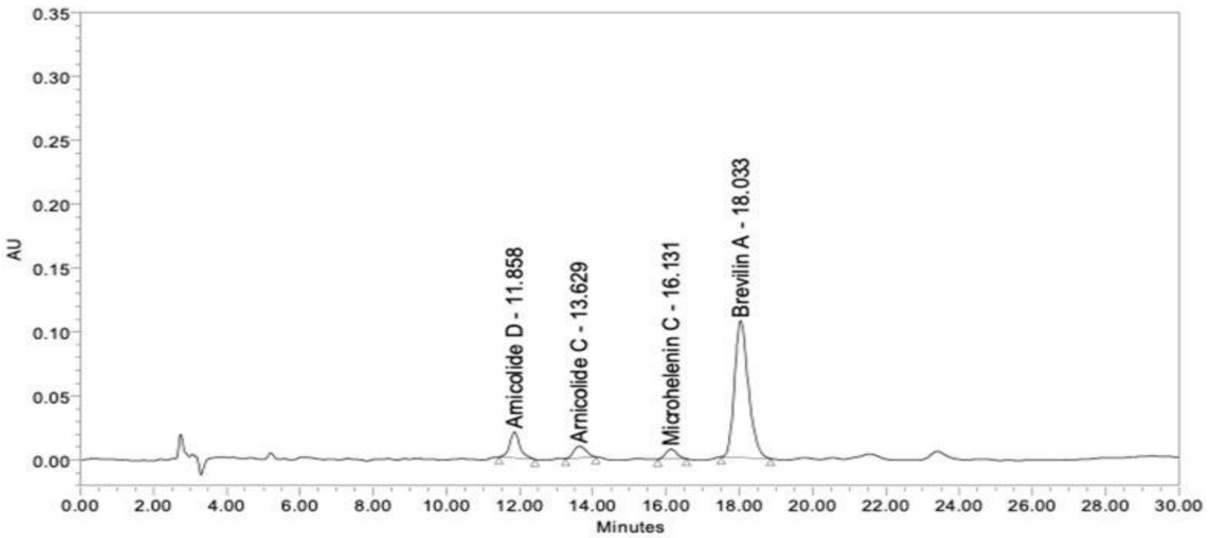

Figure S1. High-performance liquid chromatography (HPLC) chromatograms of CMXP. Extraction and analysis were performed according to previously described methods [17]. The chromatogram was reproduced with permission under the Creative Attribution License (CC BY 4.0 license).
